# Pericardial fluid cytology as a diagnostic tool for diffuse large B-cell lymphoma: a case report

**DOI:** 10.1515/almed-2025-0107

**Published:** 2025-10-03

**Authors:** Antonio Sierra-Rivera, Clara Ramírez-Serra, Laura Sahuquillo-Frias

**Affiliations:** Department of Clinical Analysis, Valencia General University Hospital, Valencia, Spain; Clinical Biochemistry Research Group, Vall d’Hebron Research Institute (VHIR), Biochemical Core Facilities, Vall d’Hebron University Hospital, Autonomous University Barcelona, Barcelona, Spain

**Keywords:** cytological study, diffuse large B-cell lymphoma, emergency laboratory, laboratory professional, pericardial fluid, primary cardiac lymphoma

## Abstract

**Objectives:**

Primary cardiac lymphoma represents an extremely rare entity of extranodal non-Hodgkin lymphoma.

**Case presentation:**

We report the case of a woman who presented with dyspnea, asthenia, chest pain, cough, and odynophagia. Investigations revealed a diffuse large B-cell lymphomas type primary cardiac lymphoma through cytological examination of pericardial fluid. Confirmatory diagnosis was established through cytology of pericardial effusion, supported by imaging techniques. The patient completed all cycles of chemotherapy and is currently in remission.

**Conclusions:**

This case highlights the essential role of accurate cytological evaluation by laboratory professionals, which can facilitate early diagnosis and may obviate the need for more invasive procedures such as endomyocardial biopsy.

## Introduction

Primary cardiac lymphoma (PCL) is a rare type of non-Hodgkin lymphoma that involves the heart, the pericardium, or presents as a large tumor mass located in the heart, most frequently found in the right chambers [1]. The clinical manifestations are nonspecific, including heart failure, precordial pain, arrhythmias, and cardiac tamponade, and will depend on the location and extent of the tumor, often leading to a late diagnosis and worsening prognosis of the disease [2]. Imaging techniques such as transesophageal echocardiography (TE), computed axial tomography (CAT) and magnetic resonance imaging (MRI) are crucial for diagnosing PCL. However, confirmatory diagnosis is established through endomyocardial biopsy or cytology of pericardial or pleural effusion [3].

Herein, we present the case of a woman diagnosed of diffuse large B-cell lymphomas (DLBCL) type PCL through cytological examination of pericardial fluid (PF).

## Case presentation

All procedures performed in this study were in accordance with the ethical standards of the Institutional and/or National Research Committee, as well as with the 1964 Helsinki Declaration and its later amendments, or comparable ethical standards. Written informed consent was obtained for the publication and presentation of clinical images.

A 55-year-old woman presented to the emergency department after being referred from her primary care center for evaluation of a left supraclavicular adenopathy. She reported experiencing symptoms of dyspnea, asthenia, chest pain, cough, and odynophagia for the past six days.

Her medical history included a thyroidectomy for follicular thyroid carcinoma, performed five years ago, as well as hypertension, obesity, and bronchial asthma. The patient had been smoking 10 cigarettes per day since the age of 17 and had no history of alcohol consumption.

The initial examination revealed a weight of 96.4 kg, a body temperature of 36 °C, an oxygen saturation of 96 %, a heart rate of 95 beats per minute, a blood pressure of 136/81 mmHg, urine output of 180 cc/2 h, and tachypnea at 24 breaths per minute. Pulmonary auscultation showed a preserved vesicular murmur with occasional wheezing and minimal bibasilar crackles. The electrocardiogram demonstrated sinus tachycardia at 100 bpm with a PR interval of 0.16 s, a narrow QRS complex at 60° with an R>s pattern in V4 and a flattened T wave in V6 with a ventricular extrasystole with an inferior axis.

A complete blood workup and a positron emission tomography-computed axial tomography (PET-CAT) scan were requested.

Blood tests revealed a hemoglobin level of 110 g/L, an erythrocyte sedimentation rate of 84 mm/h, a gamma-glutamyl transferase of 79 U/L, a lactate dehydrogenase (LDH) of 453 U/L and a C-reactive protein of 7.10 mg/dL. The rest of results for blood count, coagulation, biochemistry, hormones, tumor markers, protein electrophoresis were within normal ranges and are detailed in Table 1.

**Table 1:** Results of blood count, coagulation, biochemistry, hormones, tumor markers and proteinogram.

Parameter	Result	Units	Reference intervals
**Hemogram**			

Red blood cells	3.91^a^	10^12^/L	4–5.2
Hemoglobin	110^a^	g/L	120–150
Hematocrit	0.361	L/L	0.360–0.450
Mean corpuscular volume	92.3	fL	80–98
White blood cells	8.27	10^9^/L	4–11
Neutrophils	5.3	10^9^/L	2–7
Lymphocytes	2	10^9^/L	1.2–3.5
Monocytes	0.8	10^9^/L	0.1–1
Eosinophils	0.2	10^9^/L	0–0.5
Basophils	0	10^9^/L	0–0.2
Platelets	248	10^9^/L	140–400

**Coagulation**			

Prothrombin time	14.1	second	8.6–16
INR	1.14		0.70–1.30
Activated partial thromboplastin time	31.9	second	24.4–39.7
Fibrinogen	4.83	g/L	2.39–6.1

**Biochemistry and hormones**			

Glucose	98	mg/dL	74–110
Urea	29	mg/dL	17–43
Creatinine	0.65	mg/dL	0.51–0.95
Alanine aminotransferase (ALT)	28	UI/L	7–35
Aspartate aminotransferase (AST)	21	UI/L	10–35
Bilirubin	0.57	mg/dL	0.30–1.20
Alkaline phosphatase (AP)	118	UI/L	30–120
γ-Glutamyl transferase (GGT)	79^a^	UI/L	6–38
Lactate dehydrogenase (LDH)	453^a^	UI/L	0–248
C-reactive protein (CRP)	7.10^a^	mg/dL	0.03–0.50
Erythrocyte sedimentation rate (ESR)	84^a^	mm/h	0–20
Iron	30^a^	µg/dL	50–150
Ferritin	149	ng/mL	25–250
Folic acid	3.8	ng/mL	2.9–16.9
Vitamin B_12_	542	pg/mL	211–911
Thyrotropin (TSH)	6.713^a^	mU/L	0.550–4.780
Triiodothyronine (T3)	2.22^a^	pg/mL	2.30–4.20
Thyroxine free (FT4)	1.03	ng/dL	0.80–1.76
Anti-TSH receptor antibodies (TRAbs)	<0.3	UI/L	0–1.8
Anti-thyroglobulin antibodies (TgAbs)	14	UI/mL	0–115
Anti-thyroperoxidase antibodies (TPOAb)	<28	U/mL	0–60

**Tumor markers**			

Carcinoembryonic antigen (CEA)	1.5	ng/mL	0–3.5
Carbohydrate antigen 19.9 (CA 19.9)	10.8	U/mL	0–37
Carbohydrate antigen 125 (CA 125)	13	U/mL	0–35
Carbohydrate antigen 15.3 (CA 15.3)	5.7	U/mL	0–35
Squamous cell carcinoma antigen (SCCAg)	0.7	ng/mL	0–2.3
β2-Microglobulin	1.6	mg/L	0.7–2.5
α-Fetoprotein	1.7	ng/mL	0–7
Neuron-specific enolase (NSE)	17.6	ng/mL	0–18

**Protein electrophoresis**			

Albumins	4	g/dL	3.5–5.2
α1-Globulins	0.44^a^	g/dL	0.21–0.35
α2-Globulins	0.93^a^	g/dL	0.51–0.85
β1-Globulins	0.42	g/dL	0.34–0.52
β2-Globulins	0.36	g/dL	0.23–0.47
γ-Globulins	0.84	g/dL	0.8–1.35

^a^Values outside normal ranges.

The PET-CAT scan revealed multiple adenopathies with an anterior mediastinal mass suggestive of a lymphoproliferative process. Additionally, a significant pericardial effusion was observed, predominantly in the posterior sac, measuring up to 25 mm.

Given the confirmation of a severe, circumferential pericardial effusion without clinical or analytical signs of cardiac tamponade, the decision was made to perform evacuative pericardiocentesis. A total of 650 cc of serous fluid was obtained. Following the procedure, the patient experienced significant clinical improvement. The fluid was sent to the emergency laboratory for biochemical and cytological analysis.

**Figure 1:**
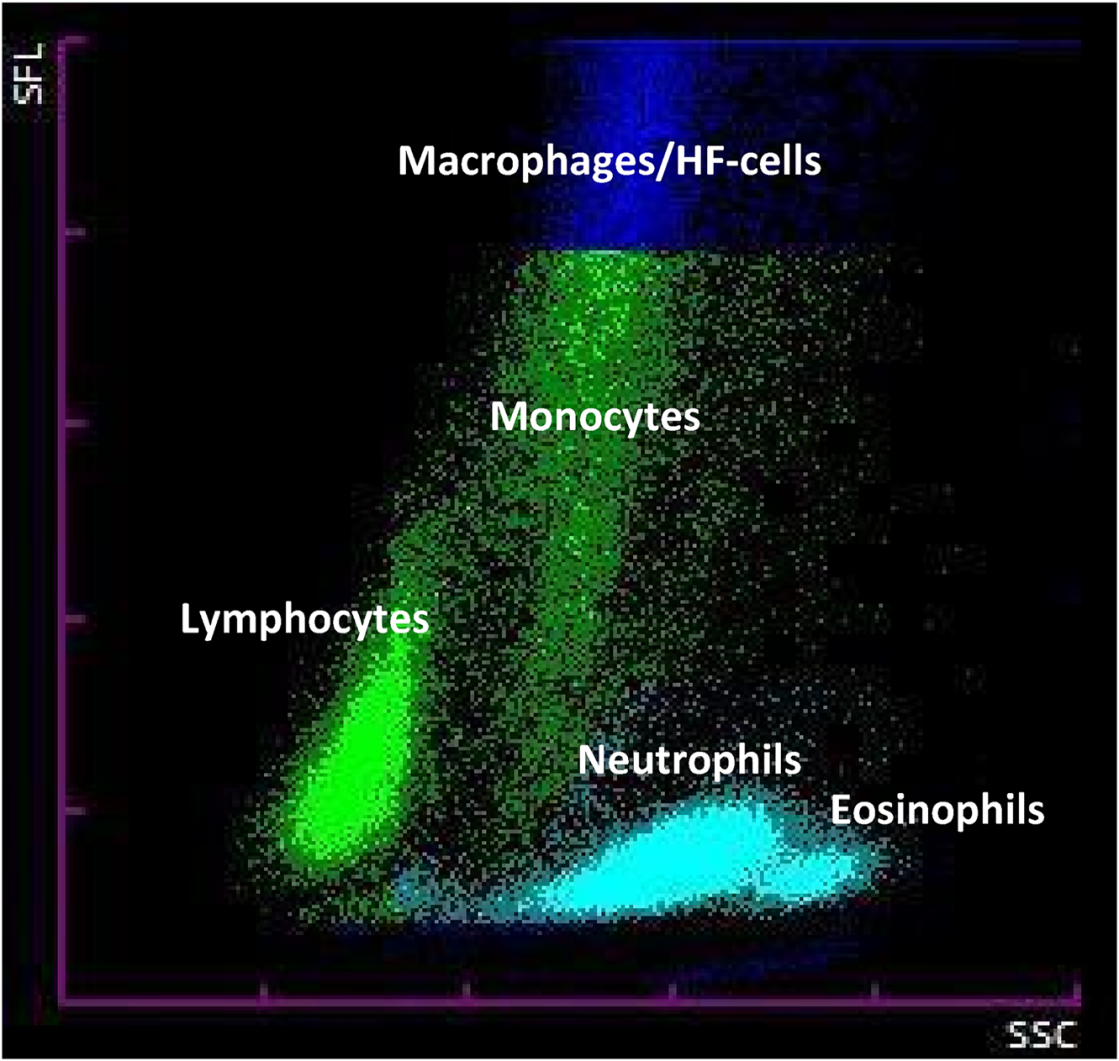
Scattergram showing the different well-differentiated cell subpopulations, including a population area in the high fluorescence zone corresponding to a subpopulation of cells that could be identified as atypical cells of hematologic origin. HF, high fluorescence.

Biochemical analysis of the PF was performed using the AU5800 analyzer (Beckman Coulter^®^), yielding the following results: total proteins of 3.7 g/dL (<3 g/dL suggestive of transudate), adenosine desaminase of 26 U/L (>45 U/L suggestive of tuberculosis), glucose 85 mg/dL (>60 mg/dL suggestive of transudate) and LDH 549 U/L (<165 U/L suggestive of transudate). For automated cytological analysis, the Sysmex XN-1000 flow cytometer (Sysmex^®^) was used, yielding the following results: a nucleated cell count of 6,840 cells/µL and 2000 erythrocytes/µL. Additionally, the scattergram demonstrated well-differentiated cell subpopulations. However, it highlighted the presence of a high fluorescence population accounting for 36.7 % of the total cells, which required further review under a light microscope (Figure 1).

**Figure 2:**
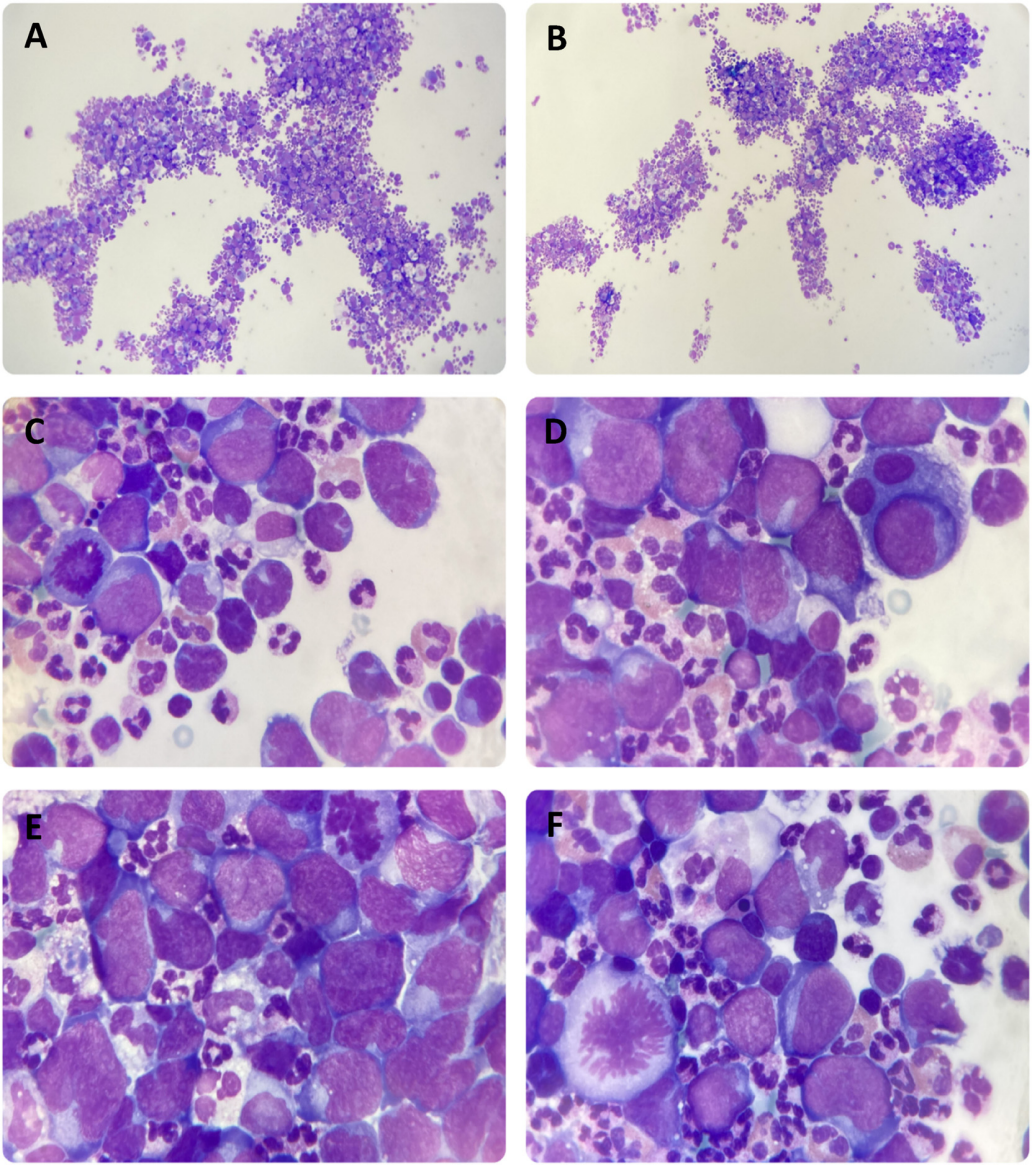
Cytology of the pericardial fluid, after centrifugation and May-Grünwald Giemsa staining, showed a predominance of neutrophils with a minority presence of lymphocytes and eosinophils (A-B, original magnification × 10). A leukocyte differential count could not be performed due to the high presence of atypical cells of hematologic origin. These cells were described as large, with a high nucleus-cytoplasm ratio, lax chromatin, visible nucleoli, irregularly contoured nuclei, highly basophilic cytoplasm and accompanied by abundant mitoses (C-F, original magnification × 100).

Cell differentiation was performed under a light microscope through cytocentrifugation of the PF and May-Grünwald Giemsa staining. Neutrophils predominated, with a minor presence of lymphocytes and eosinophils, as indicated by the scattergram. However, the high fluorescence cell population corresponded to atypical cells of possible hematologic origin. These cells were described as large, with a high nucleus-to-cytoplasm ratio, lax chromatin, visible nucleoli, irregular nuclear contours, highly basophilic cytoplasm and accompanied by abundant mitoses (Figure 2). The emergency laboratory was contacted and recommended performing an immunophenotyping and anatomopathological study to confirm the presence of neoplastic disease and characterize the pathology. The pathological study, based on cytological analysis of PF, confirmed the presence of malignant cells compatible with non-Hodgkin lymphoma. Immunohistochemical analysis of the lymph node biopsy revealed positivity for CD20, MUM1, BCL2, and weak positivity for BCL6, while CD5, CD10, CD30, and MYC (<30 %) were negative. Ki67 expression was 80–90 %.

The bone marrow biopsy was normocellular and showed no evidence of infiltration by a lymphoproliferative process. Biochemical and cytological study of the cerebrospinal fluid yielded normal results: glucose 82 mg/dL [RI: 38–82 mg/dL]; proteins 18 mg/dL [RI: 15–45 mg/dL]; 0 nucleated cells/µL and 5 erythrocytes/µL, indicating the absence of central nervous system infiltration. Based on these results, the case was classified as DLBCL with a bulky mediastinal mass and a non-germinal centre phenotype in stage IV-B.

Finally, the patient was initiated on combination chemotherapy with R-CHOP (rituximab, cyclophosphamide, hydroxydaunorubicin, oncovin, and prednisone), along with prophylactic rasburicase due to the high risk of developing a tumor lysis syndrome.

## Discussion

Primary cardiac tumors have a prevalence of 0.02 % and are classified as either benign or malignant tumors, with benign tumors accounting for over 75 %, the most common being myxomas [4], [5], [6]. The prevalence of PCL among cardiac tumors is 1–2 % [7], [8], [9]. Moreover, it should be noted that more than half of cardiac lymphomas are identified as DLBCL of high malignancy grade, with a tendency for rapid progression [10], 11]. Approximately 80 % of PCL cases in immunocompetent individuals correspond to DLBCL [12]. PCL must be distinguished from secondary cardiac involvement in disseminated lymphomas, which belong to the classification of non-Hodgkin blood cancers. It should also be differentiated from other primary malignant cardiac tumors, such as angiosarcomas, the most common primary malignant neoplasm of the heart, as well as from more frequently occurring benign cardiac tumors, such as myxomas and lipomas.

It is noteworthy that DLBCL are extremely fast-growing neoplasms. Their diagnosis is difficult and remains challenging due to the clinical manifestations are highly variable and nonspecific, which may include B symptoms (fever, weight loss, and night sweats), chest pain, dyspnea, and, in rare cases, superior vena cava obstruction [13]. In addition, DLBCL can also mimic common presentations of heart failure, arrhythmia and pericarditis [11], 14].

The most common causes of death in patients with PCL are lymphoma progression, cardiac arrhythmias, intractable heart failure, and sepsis [13]. The prognosis of PCL remains poor if left untreated, primarily due to late diagnosis and the aggressive nature of the disease. The median survival is approximately 12 months [13]. Therefore, given that DLBCLs are highly malignant and aggressive neoplasms, early diagnosis and prompt initiation of treatment, typically chemotherapy and, in some cases, surgery, are key factors to improving morbidity and mortality outcomes, ultimately increasing survival rates. The problem is that most patients remain asymptomatic in the early stages, often leading to delayed diagnosis and advanced disease at presentation [11], 14].

Imaging techniques such as TE, MRI, positron emission tomography (PET) or the combined PET-CAT are fundamental for diagnosing PCL [1]. However, an endomyocardial biopsy of the tumor or cytological examination of pericardial or pleural effusion is required to confirm the diagnosis [3]. Pathological diagnosis of cardiac involvement before chemotherapy is essential to guide treatment. Therefore, it is crucial to send the PF to the laboratory for cytological analysis to confirm malignant involvement [15]. In this case, if the cytological study had been inconclusive, or pericardiocentesis had been impossible, the next step would have been image-guided transvenous pericardial or epicardial biopsy, which is a more invasive procedure [1]. Although the prognosis for these patients is unfavorable, early diagnosis and appropriate treatment can significantly improve survival.

## Conclusions

With this case, we aim to emphasize that, despite the importance of imaging techniques, the proper training of laboratory professionals plays a crucial role in performing a thorough cytological study. This, along with the rapid response time of the emergency laboratory, contributes to early diagnostic confirmation. In this case, the combined morphological, immunohistochemical, and cytogenetic findings supported the diagnosis of DLBCL with a bulky mediastinal mass and non-germinal centre phenotype that debuted with dyspnea due to tamponade by infiltrative pericardial effusion and was diagnosed *de novo* through the PF cytological study conducted by the laboratory professional.

## Lessons learned

– Primary cardiac lymphoma, though rare, should be considered in patients with unexplained pericardial effusion and systemic symptoms.
– Cytological analysis of pericardial fluid can provide a rapid, minimally invasive diagnostic alternative to endomyocardial biopsy for diffuse large B-cell lymphoma.
– Flow cytometry and careful microscopic evaluation of pericardial fluid can detect atypical lymphoid populations suggestive of malignancy.
– Early diagnosis through cytological analysis may improve prognosis by facilitating prompt initiation of appropriate chemotherapy.
– Timely collaboration between clinicians and laboratory professionals is essential for early recognition and management of aggressive lymphomas.

## References

[1] Ellen S, Emma H (2023). Primary cardiac lymphoma: a case report. Eur Heart J Case Rep.

[2] Porcar Ramells C, Clemente González C, García Parés D, Guardia Sánchez R, Pérez Ayuso M, García-Bragado Dalmau F (2002). Linfoma cardíaco primario: diagnóstico citológico y tratamiento con respuesta a poliquimioterapia y autotrasplante de precursores hematopoyéticos. Presentación de un caso y revisión de la literatura. An Med Interna.

[3] Cioc AM, Jessurun J, Vercellotti GM, Pambuccian SE (2014). De novo CD5-positive primary cardiac diffuse large B-cell lymphoma diagnosed by pleural fluid cytology. Diagn Cytopathol.

[4] Odim J, Reehal V, Laks H, Mehta U, Fishbein MC (2003). Surgical pathology of cardiac tumors. Two decades at an urban institution. Cardiovasc Pathol.

[5] Kamiya H, Yasuda T, Nagamine H, Sakakibara N, Nishida S, Kawasuji M (2001). Surgical treatment of primary cardiac tumors: 28 years’ experience in Kanazawa university hospital. Jpn Circ J.

[6] Gabe ED, Rodríguez Correa C, Vigliano C, San Martino J, Wisner JN, González P (2002). Mixomas cardíacos: correlaciónanatomoclínica. Rev Esp Cardiol.

[7] Reynen K (1996). Frequency of primary tumors of the heart. Am J Cardiol.

[8] Miguel CE, Bestetti RB (2011). Primary cardiac lymphoma. Int J Cardiol.

[9] Sultan I, Aranda-Michel E, Habertheuer A, Kilic A, Arnaoutakis G, Bianco V (2020). Long-term outcomes of primary cardiac lymphoma. Circulation.

[10] Chen H, Qian S, Shi P, Liu L, Yang F (2020). A presentation, treatment, and survival analysis of primary cardiac lymphoma cases reported from 2009 to 2019. Int J Hematol.

[11] Bussani R, De-Giorgio F, Abbate A, Silvestri F (2007). Cardiac metastases. J Clin Pathol.

[12] Gowda RM, Khan IA (2003). Clinical perspectives of primary cardiac lymphoma. Angiology.

[13] Petrich A, Cho SI, Billett H (2011). Primary cardiac lymphoma: an analysis of presentation, treatment, and outcome patterns. Cancer.

[14] Gordon MJ, Danilova O, Spurgeon S, Danilov AV (2016). Cardiac non-hodgkin’s lymphoma: clinical characteristics and trends in survival. Eur J Haematol.

[15] Adler Y, Charron P, Imazio M, Badano L, Barón-Esquivias G, Bogaert J (2015). ESC guidelines for the diagnosis and management of pericardial diseases: the task force for the diagnosis and management of pericardial diseases of the European society of cardiology (ESC) endorsed by: the European association for cardio-thoracic surgery (EACTS). Eur Heart J.

